# Singing Away the Social Distancing Blues: Art Therapy in a Time of Coronavirus

**DOI:** 10.1177/0022167820927807

**Published:** 2020-09

**Authors:** Nisha Gupta

**Affiliations:** 1 University of West Georgia, Carrollton, GA, USA

**Keywords:** coronavirus, art therapy, music therapy, cinematherapy, expressive art therapies, solidarity, community crisis

## Abstract

This essay explores the abundance of art flourishing as a therapeutic antidote to the COVID-19 pandemic and panic arising across the world. Specifically, I discuss how the act of viewing, making, and sharing music, street art, paintings, graphic art, cinema, and digital videos can serve as a therapeutic vehicle for empowerment, solidarity, and collective action as most human beings strive to adopt practices of extreme social distancing as the recommended community mitigation strategy to help save lives before a vaccine is developed. This essay explores how therapeutic art-making can promote physical, mental, and social health at a time in history when all of these are under threat by COVID-19. I root these claims in theoretical literature from art therapy, as well as in inspiring and heart-warming examples of the beautiful coronavirus art that has already begun to fill our digital landscape with motivation, resiliency, and hope, though the crisis is still in its early stages.

Creativity puts toxins to good use.

—Shaun McNiff

It is March 17^th^, 2020, and I have been bunkered inside my studio apartment for 5 days. I am practicing extreme social distancing to do my humble part in protecting the dear lives of my human community from COVID-19. Ten cotton canvases and a basket full of acrylic paints sit beside me to companion me through these turbulent weeks or months, as the world implodes outside my studio walls. It is apparently still early in the United States’ crisis of coronavirus; our nationwide tsunami is yet to arrive but certainly only days away. *This is just the beginning*, as the doctors say. And yet already the artists have answered the rally-cry, pouring their beauty onto social media posts, across street buildings, and through apartment windows to connect us, soothe us, and inspire collective action.

Shaun McNiff (1981, p. v) describes art therapy as one of the most “ancient forms of healing,” affirming the primal human need to embrace the arts as medicine for the soul amid personal and collective struggle. According to McNiff, engaging in the arts is empowering during perilous life circumstances, because it reminds us of the powerful will of the human spirit to remake and transform both internal and external realities. Art empowers us to *participate*, having faith in our ability to make a difference:

throughout time, art has shown that it can change, renew, and revalue the existing order. If art cannot physically eliminate the struggle of our lives, it can give significance and new meaning and a sense of active participation in the life process. (McNiff, 1981, p. vi)

In response to the helplessness, uncertainty, and fear caused by the new coronavirus, health care professionals are beckoning ordinary citizens to tap into our personal power and realize we can play an active role in changing the existing order to reduce the impending death toll of this global health crisis. They urge us to participate in the community mitigation strategy of “social distancing” to help save lives, which means staying home and physically distancing from others as much as possible to avoid contracting and spreading the infection at a rate which would overwhelm the health care system, thereby leading to more lives lost.

While social distancing is empowering in theory, it can also feel disempowering as a psychological reality due to the loneliness, restlessness, and panic that arises as the days slog slowly and uncertainly. The expressive arts provide a supplementary, empowering antidote to this crisis of health. Art is available for all people to participate in as a tried-and-true “vaccine” of sorts, working its therapeutic magic to protect the physical, social, and mental health of the human species as we struggle together to confront COVID-19 with simultaneous distance and solidarity. This essay honors therapeutic expressions of art that have already begun to imbue our world with hope amid this global pandemic. In writing this essay so early into the outbreak, I predict these examples to be but a fraction of the collective artistic awakening to soon unfold—perhaps at a comparable rate, and with equal force, as the spread of coronavirus.

## Social Distancing Solidarity Via Song

One mental health consequence of social distancing is the terrible loneliness that can prevail, as people are necessitated to remain inside our homes and away from the communal comradery of our daily lives, which we may have taken for granted until now. Our worlds can feel eerily quiet and small when mandated to work and school from home, avoid restaurants and bars, and stay at a six-foot distance from others for weeks or months to come. Physical distancing can certainly be experienced as a psychological distancing without intentional efforts to offset the isolation. Health care professionals are well aware of these mental health consequences; they encourage frequent virtual check-ins with one another—particularly those that are most vulnerable to COVID-19, such as our beloved elders—to preserve our sense of intimate community bonding in this time of self-quarantining.

As a therapeutic form of art, music offers that bonding power. Historically, music has been used by people to remain motivated, resilient, and united in the face of collective challenge, from the *Sea Shantis* that sailors in the 18th century chanted together as they labored to weather harsh ocean tides, to the freedom songs sung by the Black community in the 20th century amid the tiring fight for civil rights. Tracey Nicholls (2014) writes about music’s emotional power to join people in solidarity and build movements together:

it is because of the way music feeds into our emotional lives and because of the sense of social well-being we get from sharing emotional states with others that music so frequently accompanies movements that build, and depend upon, solidarity.

Whereby solidarity is defined as “the harmony of interests and responsibilities among individuals in a group, especially as manifested in unanimous support and collective action for something” (Laurence & Urbain, 2011, p. 5), musical solidarity has the therapeutic ability to puncture the isolation of social distancing, to foster resiliency by lifting the collective spirit, and to move people’s emotions toward spirited *action—*even if that action, in these circumstances, means staying home to save the lives of others. Music can also assuage the stress inflicted by social distancing and the COVID-19 pandemic by soothing the autonomic nervous system through rhythm and melody (Thoma et al., 2013).

In this time of coronavirus, examples of therapeutic music-making unceasingly flow. As a psychology professor who teaches art therapy, my undergraduate students and I were supposed to do a music therapy lesson last week and engage in an improvisational jam-session together in the classroom. Each student was invited to bring an object that can serve as an instrument, be it a guitar or harmonica or spoons or their voice. To comply with the social distancing mitigation strategy recommended by health care organizations, we decided to move the class online via “Zoom meetings” as a last-minute plan. Yet social distancing did not impede our musical solidarity. Instead, the 20 students and I experimented with a virtual, improvisational, jam session to join our spirits in a fun rhythmic flow. Of course, the rhythm was slightly delayed because of the Internet’s lag-time. Of course, our harmony could not find perfect synchronization through our laptops and phones. Yet still we sang together, clapped together, strummed together, and laughed together, accessing a primal mode of communal being-together through collective sound making across our individual screens. Our improvisational virtual jam session brought us enough joy and love to overpower the terror of COVID-19 for just a little while! But the next day I awoke to something all the more moving: one of the most poignant demonstrates of musical solidarity I have ever seen. A video was circulating of the people in Italy, who are currently undergoing a national quarantine because of the rapid, uncontainable spread of the infection across their nation at a capacity that far overwhelms their health care capacities and has led to dire life-and-death decisions in the hospitals (with warning that the United States may follow suit if we do not social distance *now*). The situation in Italy is tragic; there can be no denying the collective trauma among COVID-19 patients and health care providers and self-isolated citizens alike. And yet this video of the Italians conveys unbridled joy (viewable at https://www.youtube.com/watch?v=8r357UgH7hU). The video captures them congregating on the balconies of their apartments, strumming and shaking whatever instruments are available to them, and joining the voices to sing together into the wide open air. They are laughing, dancing, and clapping in celebration of one another. It brings the viewer to tears to witness such a delightful display of community; it brings me close to tears to write this even now. The antidote of musical solidarity in a time of coronavirus provides a joyful reminder of the deep human will to always find our way back to one another. Even a nationwide quarantine or a deadly pandemic cannot prevent us from connecting and supporting each other through life’s darkest hours. Since this video of the Italians’ impromptu concert went viral, social media has become abundant with musicians generously sharing “virtual concerts” for the general public to watch from the safety of their homes, such as members of the Atlanta Symphony Orchestra, John Legend, Coldplay, and many of my musical friends on Facebook. In the days to come, I imagine these bonds of music will exponentially grow to remind each of us that we are not alone.

## Imaging the Virus Via the Visual Arts

One challenge in mobilizing the masses to understand the severity of threat of COVID-19 and the importance of social distancing is the invisibility of the disease. Our eyes cannot literally *see* how the coronavirus spreads from person to person. In the United States, at the time of writing this essay, many of us can also not yet *see* the tragic effect of the disease on our community members or loved ones, with the exception of the major epicenters of the outbreak such as Washington and New York. At this point, the pandemic remains shrouded in invisibility to most American eyes, lest we find the courage to watch news clips of the traumatic hospital scenarios in China and Italy. Similar to a psychological understanding of climate change apathy, it is difficult to hold tangible concern for a future calamity that appears intangible to us in present day. Thus, health care professionals’ pleas for American citizens to take seriously the adoption of health precautions and social distancing practices can fall on apathetic ears and eyes. What can help people reverse apathy and confront reality is the restoration of vision—of literally *seeing* the coronavirus and its impact on us and our loved ones: “vision is . . . one of the means by which we interact with and relate to the world, and by increasing our visual awareness, we extend and intensify our relationship to life” (McNiff, 1981, p. 155).

As a therapeutic vehicle, the visual arts help make the unconscious conscious. Creating an image brings tangible form to psychological realities, which remain typically unseen to the human eye, thereby allowing us to become aware of and confront these realities. During crisis experiences that are emotionally difficult to confront such as the threat of COVID-19, an image can act as an external container within which to “place” our inner disturbances so we can safely understand and work through them. As McNiff (1998) states,

The art of placing a troublesome experience or thought into a creative space we have made literally changes its place within our lives. The artistic act will often have a corresponding effect on our overall relationship with the disturbance . . . when we use our disturbances as materials of expression we see that everything in life is fuel for the creative process. (p. 74)

After making visual artwork, we can then examine our image at a safe distance so a new perspective can be discovered, which allows our feelings and relationship to the crisis to transform. As tangible products, our artwork can also be shared with others, inviting them to dialogue with our image, discover personal meanings in it, and emotionally connect to it as shared human experience; thus visual artwork can offer collective healing. Finally, visual artworks can have an everlasting and historical quality, behaving as tangible memorials that forever commemorate this present moment in our personal and collective histories (Shulman & Watkins, 2008). Memorial making during times of collective crisis can bring healing.

Prior to most countries enforcing or strongly encouraging social distancing, it is amazing to witness the coronavirus street art that sprung up as if overnight. In London, street artist Pegasus painted a mural on a street corner featuring Prime Minister Boris Johnson urging people to stop panic-buying supplies in grocery stores as they prepare to social distance, he wanted to remind them to be considerate to others during this crisis. In Rome, street artist Laika painted a mural above a popular restaurant to address the ignorance and xenophobia toward Chinese people in Italy as a result of COVID-19; the image includes a Chinese woman dressed in surgical attire and a face mask with a speech bubble that reads: “There’s an epidemic of ignorance going around . . . we must protect ourselves!” In Naples, artist Nello Petrucci erected a giant street mural of the *Simpsons* family wearing face masks on their couch with the words “STAY HOME!” spray-painted onto the image. From within their homes, adults and children are transforming boredom into creativity by crafting their own images to share with the public. In Italy, many quarantined families hang colorful, painted banners outside their balconies with words of encouragement such as: “andra tutto bene” meaning “everything will be fine.” In the United States, where a national quarantine has not (yet) been administered, the visual arts are embraced to make the invisible visible, remind Americans of the gravity of the situation, and offer encouragement and precaution. I painted an acrylic painting called “Social Distancing Blues,” which visualizes the coronavirus hovering across the sky in bright red paint outside my apartment windows. I shared the image on social media as a public service advertisement with the message: “Even though the virus looks pretty from afar, don’t trust it! Stay at home, folks!” A few days later my mother, a senior citizen who is in the high-risk category of COVID-19, was inspired to create her own graphic image as a public service announcement. She used the phone app Bitmoji to create a comic strip of herself as a cartoon character social distancing. In the comic strip, she offers healing greetings and safety strategies to help others cope with COVID-19: “Good day.” “Thinking of you all.” “Are you ok?” “I am social distancing cuz I want to listen to the CDC advice and so should you,” “Wash your hands!” “Don’t touch your face!” “Nature walks,” “healing through the Internet,” “No peeking at the 401k,” “Hang in there!” “Don’t panic,” “We can do it!” What is so moving about these examples is that in the process of making and sharing coronavirus art, a shift seems to occur where we become empowered *to do something* about the COVID-19 crisis. We seem to be creating images to help slow the virus down, spread reminders of kindness, and nurture collective resiliency. Just as McNiff suggested that placing a troublesome experience into an artistic container can transform our relationship to it, making visual art about coronavirus and social distancing can help transform collective anxiety into collective support and action. Thus, this time in history may forever be memorialized as a moment of precious human solidarity.


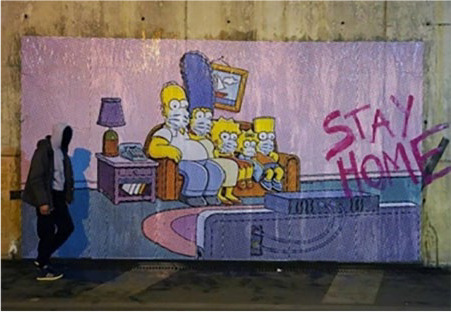



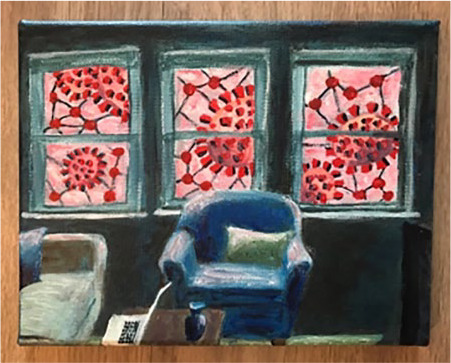



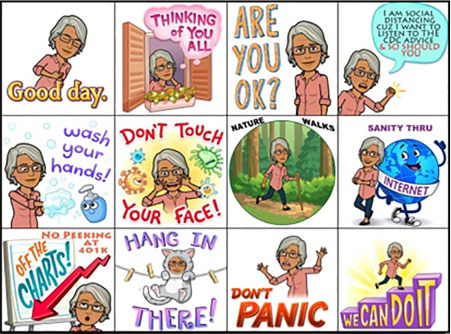


## Coronavirus Catharsis and Containment Via Cinema

One stressful aspect of coronavirus is that it is a new pandemic we do not know much about; our understanding is evolving day by day. Moreover, mixed messages are being disseminated from politicians, health care professionals, organizational leaders, and our intimate social circles. This lack of information and mixed messaging breeds anxiety by making us feel out of control and unsure of what to believe. The future feels unknown and we struggle to find a secure leg on which to stand, grasping for some semblance of certitude with which to anticipate a narrative about the impending days, weeks, and months to come.

Cinema is increasingly embraced as a form of expressive art therapy, because movies offer relatable narratives with characters and plotlines that can express the shared human experience. *Cinematherapy* is the intentional use of movie viewing to gain self-insight and psychological transformation, as audiences’ emotions and perspectives transform while watching a movie (Berg-Cross et al., 1990; Sharp et al., 2002). Most films offer narratives with a reliable temporal structural—a *beginning* in which a conflict emerges, a *middle* during which the crisis is grappled with, and an *ending* during which the conflict is resolved. If we watch a movie whose crisis is similar to our own, we can find incredible catharsis and relief by witnessing a resolution to our crisis offered by the movie’s storyline. Moreover, because movies are a multisensory experience, they can evoke the full spectrum of emotion about a situation onscreen. Cinema’s ability to express strong emotion through sight, sound, and motion can offer emotional catharsis and containment for audiences, allowing us to externalize our own inner suffering by viewing it onscreen; the movie offers a “container” for our strong emotions. Aside from watching movies, cinematherapy also includes therapeutic filmmaking interventions: ordinary people can shoot, edit, and produce videos and films that express their difficult psychological experiences (Johnson & Alderson, 2008). It can be empowering to “control the narrative” by making a film of one’s own. Making movies helps us restore agency, gain mastery, and reduce the anxiety that corresponds with feeling out of control in crisis situations.

An interesting trend during this coronavirus crisis has been the plethora of people flocking to watch the 2011 fictional film *Contagion* on their movie streaming platforms. *Contagion* is a movie about a deadly fictional pandemic named MEV-1 whose outbreak kills 26 million people worldwide. The virus in the film kills 25% to 30% of people who catch it, as opposed to the 2% of COVID-19. Despite this significant difference, *Contagion* is nevertheless a pretty terrifying flick to watch at this time; the film gruesomely depicts the health and social impacts of a deadly pandemic. So why has it become one of the top movies streamed from Netflix and iTunes since our own pandemic broke loose—a film which appears more and more like a medical documentary than fictional thriller each day? Because despite the massive death toll that the film depicts, the movie ends with a vaccine developed which effectively immunizes human beings from MEV-1. Despite the emotional rollercoaster the audience endures as the film world explodes because of a deadly pandemic—an emotional journey that likely mirrors what we will experience as the pandemic develops in our real world—by the end of the movie, the audience can breathe a sigh of relief as the crisis is resolved and daily life returns to normal. Thus, *Contagion* offers audiences a cathartic container for our panic, a sense of control to combat uncertainty, and hope for what’s to come. Aside from the healing power of watching movies, agency and hope can also be discovered by therapeutic filmmaking in this time of coronavirus. I will again reference the magnificent art pouring from the Italians amid their current situation of enforced national quarantine. A filmmaking collective in Milan called A THING BY recently created a video compilation of Italians talking to their “former selves” from 10 days ago, which can be viewed here: https://www.youtube.com/watch?v=o_cImRzKXOs&feature=youtu.be. The Italians in the video have a dialogue with the camera of what they wish they could tell themselves 10 days ago: “Hello Daniele-from-10-days-ago. Are you scared? Nah? I’m speaking to you from the future. I know you’re busy, but wait a second. I want to update you on Italy’s latest. A huge mess is about to happen . . . ” The video showcases Italians imploring their former selves to take seriously the threat of the virus, such that they can adopt social distancing and health precautions before it is too late. While they cannot turn back time, I imagine this video was cathartic to make by providing an expressive container for feelings of regret and despair. Moreover, the Italians may regain agency and control in making this video, for it is being shared by the CDC in the United States to warn Americans of what’s to come if they make the same mistake. Through therapeutic filmmaking, the Italians may experience empowerment by helping the rest of the world avert the crisis that they themselves endured. As the pandemic spreads across the world, I imagine in-home video production will be embraced as significant therapeutic vehicle as we collectively seek to tell the story of our time.

Across the history of our species, crisis has always been intertwined with creativity. Humans are called again and again to discover and harness our primal will to create, which resides within us all, in order to survive. Existential psychologist Rollo May (1975) suggested,

If you wish to understand the psychological and spiritual temper of any historical period, you can do no better than to look long and searchingly at its art. For in the art the underlying spiritual meaning of the period is expressed directly in symbols. This is not because artists are didactic or set out to teach or to make propaganda. . . . They have the power to reveal the underlying meaning of any period precisely because the essence of art is the powerful and alive encounter between the artist and his or her world. (p. 52)

It is still early in the game, but if I am learning anything about the meaning of this time from the artists among us, it is that there is a profound desire to protect each other, sacrifice for each other, and be together-in-solidarity as an interconnected, global community when faced with this grave threat to the human species. Our lives depend quite literally on our ability to love each other—across borders, balconies, and computer screens.
